# Managing Brain Extracellular K^+^ during Neuronal Activity: The Physiological Role of the Na^+^/K^+^-ATPase Subunit Isoforms

**DOI:** 10.3389/fphys.2016.00141

**Published:** 2016-04-22

**Authors:** Brian Roland Larsen, Anca Stoica, Nanna MacAulay

**Affiliations:** Department of Neuroscience and Pharmacology, University of CopenhagenCopenhagen, Denmark

**Keywords:** K^+^ clearance, astrocytes, glutamate, brain ion homeostasis, extracellular space

## Abstract

During neuronal activity in the brain, extracellular K^+^ rises and is subsequently removed to prevent a widespread depolarization. One of the key players in regulating extracellular K^+^ is the Na^+^/K^+^-ATPase, although the relative involvement and physiological impact of the different subunit isoform compositions of the Na^+^/K^+^-ATPase remain unresolved. The various cell types in the brain serve a certain temporal contribution in the face of network activity; astrocytes respond directly to the immediate release of K^+^ from neurons, whereas the neurons themselves become the primary K^+^ absorbers as activity ends. The kinetic characteristics of the catalytic α subunit isoforms of the Na^+^/K^+^-ATPase are, partly, determined by the accessory β subunit with which they combine. The isoform combinations expressed by astrocytes and neurons, respectively, appear to be in line with the kinetic characteristics required to fulfill their distinct physiological roles in clearance of K^+^ from the extracellular space in the face of neuronal activity. Understanding the nature, impact and effects of the various Na^+^/K^+^-ATPase isoform combinations in K^+^ management in the central nervous system might reveal insights into pathological conditions such as epilepsy, migraine, and spreading depolarization following cerebral ischemia. In addition, particular neurological diseases occur as a result of mutations in the α2- (familial hemiplegic migraine type 2) and α3 isoforms (rapid-onset dystonia parkinsonism/alternating hemiplegia of childhood). This review addresses aspects of the Na^+^/K^+^-ATPase in the regulation of extracellular K^+^ in the central nervous system as well as the related pathophysiology. Understanding the physiological setting in non-pathological tissue would provide a better understanding of the pathological events occurring during disease.

## K^+^ management in the extracellular space of the central nervous system

Neuronal activity, in the form of propagating action potentials, results in a transient release of K^+^ into the extracellular space (Frankenhaeuser and Hodgkin, 1956). Prolonged accumulation of K^+^ in the extracellular space of the central nervous system (CNS) causes wide-spread depolarization of neurons and glia which results in compromised synaptic transmission, neuronal firing, and neurotransmitter re-uptake. Consequently, it follows that extracellular K^+^ must be closely managed and therefore cleared from the extracellular space following neuronal excitation. Brain extracellular K^+^, [K^+^]_o_, has indeed proven to be tightly regulated under physiological conditions; from a basal level of ~3 mM, [K^+^]_o_ rarely rises above ~12 mM. This concentration is denoted as the ceiling level and more intense or longer electrical stimulation of neuronal pathways generally cannot push [K^+^]_o_ above this point (Krnjevic and Morris, 1972; Prince et al., 1973; Futamachi et al., 1974; Heinemann and Lux, 1977). However, typical physiologically occurring neuronal activity in the CNS is estimated to cause [K^+^]_o_ transients of only 0.2–0.4 mM above the baseline (Syková et al., 1974; Singer and Lux, 1975).

**Figure 1:**
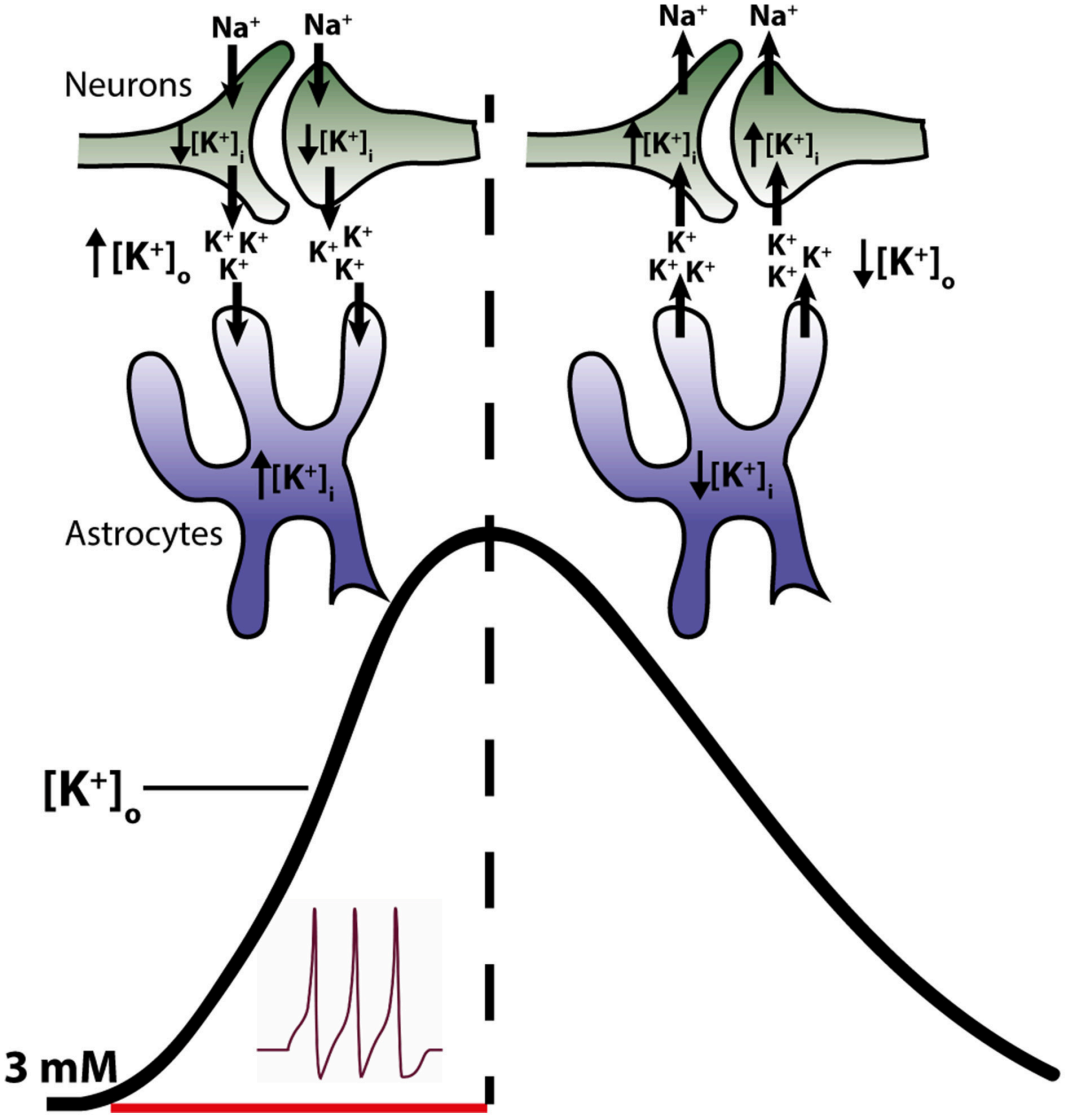
**Temporal role of astrocytes and neurons in clearance of K^+^ from the extracellular space**. Neuronal activity leads to a release of K^+^ into the extracellular space (left side of figure) resulting in a decline in intracellular K^+^ and an increase in the extracellular K^+^ (black trace in figure). Astrocytes surrounding the neurons respond to the rise in [K^+^]_o_ by increased uptake of K^+^ and consequently increase their [K^+^]_i_. Astrocytes thus act as K^+^ sinks during the initial phase of activity. Upon cessation of stimuli, K^+^ leaks out of the astrocytes, probably via Kir4.1, resulting in a reduction of astrocytic [K^+^]_i_ (right side of the figure). Meanwhile the neurons, no longer stimulated to open their voltage-gated channels, initiate reabsorbtion of their lost K^+^. Thus, in this later phase, K^+^ is directed from the astrocytes to the neurons.

**Figure 2:**
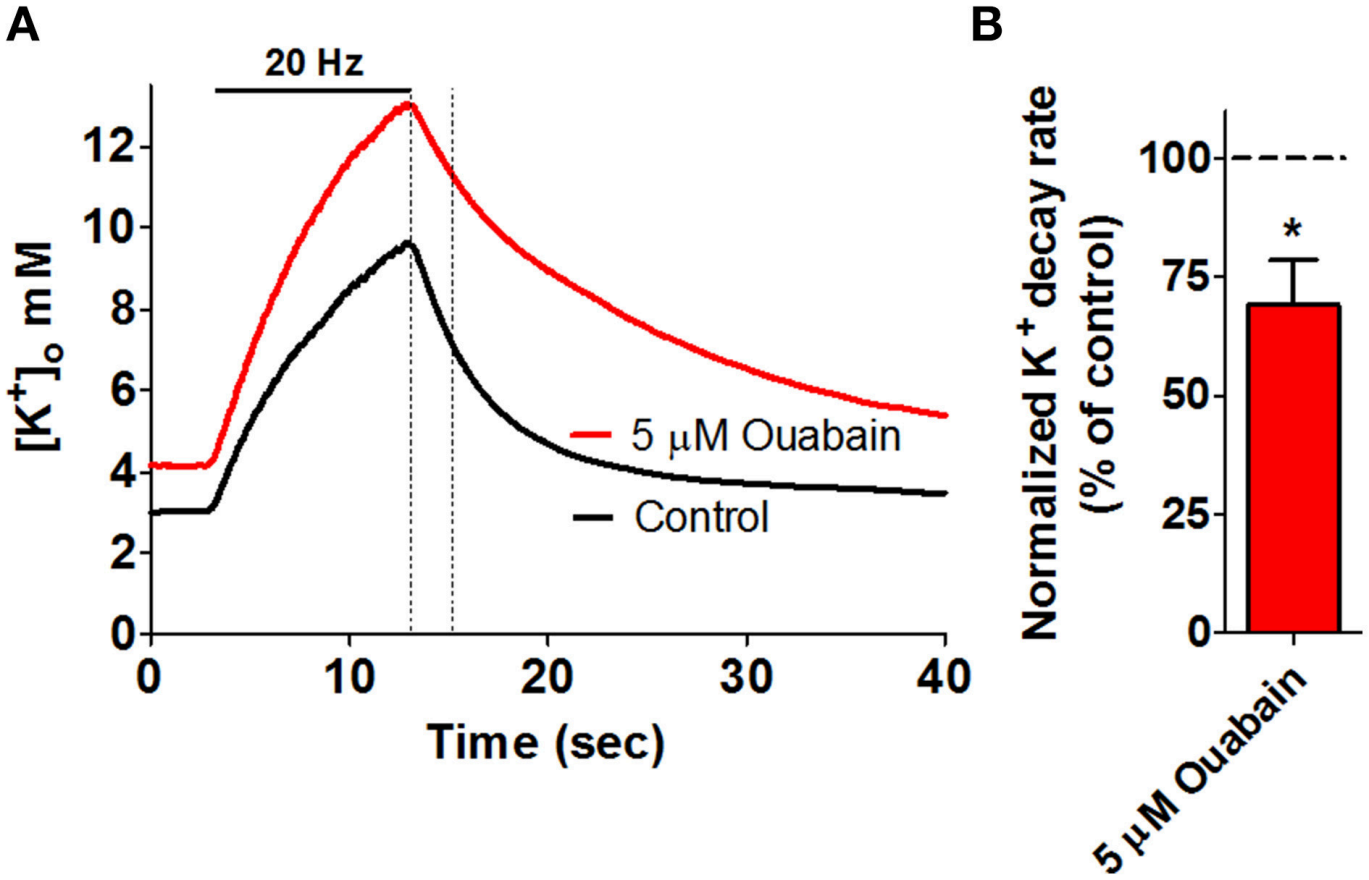
**Key role for the Na^+^/K^+^-ATPase in management of stimulus-evoked K^+^ transients**. High frequency electrical stimulation of rat hippocampal brain slices gives rise to an extracellular K^+^ transient, as measured via ion-sensitive microelectrodes. **(A)** Addition of 5 μM ouabain (red trace), inhibiting α2 and α3 Na^+^/K^+^-ATPase, results in compromised K^+^ clearance when compared to a control recording (black trace) obtained prior to addition of ouabain in the same slice. The dashed lines mark a 2-s linear segment used for quantification of the rate of K^+^-clearance and is summarized in **(B)**, ^*^P < 0.05. Figure modified from Larsen et al. (2014) with permission.

During neuronal activity, in which K^+^ is released to the extracellular space and the neuronal structures thus lose part of their intracellular K^+^, an elevation in [K^+^]_o_ is observed along with a parallel rise in intracellular [K^+^] in the neighboring glia cells (Ballanyi et al., 1987; Grafe and Ballanyi, 1987). The activity-dependent glial K^+^ accumulation points to this cellular compartment acting as a temporal K^+^ sink during neuronal release of K^+^ into the extracellular space (Ballanyi et al., 1987; Grafe and Ballanyi, 1987). Shortly following termination of neuronal activity, the glial [K^+^]_i_ declines, indicative of steady glial K^+^ efflux, while the neurons re-gain their [K^+^]_i_ via re-uptake into the neuronal structures (Ballanyi et al., 1987; Grafe and Ballanyi, 1987, see Figure 1). Surely, part of the neuronally-released K^+^ simply diffuses away in the extracellular space (Gardner-Medwin, 1983) but the molecular mechanism(s) that exercise the astrocytic clearance of extracellular K^+^ have been investigated and debated intensely ever since the original findings in the mid-1900s:

- Glial cells surrounding neurons respond directly to an increase in extracellular K^+^ by a membrane depolarization (Kuffler and Nicholis, 1964; Kuffler and Potter, 1964; Orkand et al., 1966). The fact that the glial cells in essence behaved as K^+^ electrodes, due to their high permeability for K^+^, indicated that they might act out a protective role toward neurons in connection to K^+^ by redistribution of K^+^ away from the site of activity (Orkand et al., 1966). This concept of K^+^ channel-mediated spatial buffering of activity-evoked K^+^ release relies on local differences in K^+^ equilibrium- and membrane potentials leading to Kir4.1-mediated glial K^+^ influx, electrotonic transfer of K^+^ through the gap junction-coupled glia syncytium and Kir4.1-mediated release at a distant site (Karwoski et al., 1989; Walz, 2000; Kofuji and Newman, 2004). Although the spatial buffer currents are well-documented, the quantitative contribution to extracellular K^+^ management of this channel-mediated mechanism may be limited and is under continuous debate, see (MacAulay and Zeuthen, 2012; Larsen and MacAulay, 2014; Larsen et al., 2014). This mode of K^+^ clearance will not be discussed further here.
- The Na^+^/K^+^/2Cl^−^ cotransporter of the subtype 1 (NKCC1) is functionally expressed in cultured astrocytes in which the NKCC1 is responsible for around half of the overall cellular K^+^ uptake, increasing its fractional uptake as the extracellular K^+^ increases (Walz and Hertz, 1984; Larsen et al., 2014). Taken together with its low apparent K^+^ affinity (~ 25 mM Larsen et al., 2014), which would allow it to increase its transport activity when faced with stimulus-evoked K^+^ transients, NKCC1 has been suggested as a factor involved in clearance of K^+^ from the extracellular space (Su et al., 2000; Kofuji and Newman, 2004; Hertz et al., 2015). In a recent study in rat hippocampal slices, we were nevertheless unable to demonstrate a participating role of NKCC1 in clearance of K^+^ from the extracellular space of this particular brain region (Larsen et al., 2014). As negligible transcript and protein of NKCC1 is present in astrocytes *in vivo* (Plotkin et al., 1997; Clayton et al., 1998; Zhang et al., 2014) and NKCC1 is recognized to be upregulated in cultured cells of different origin (Raat et al., 1996), NKCC1 appears to be active in cultured astrocytes but not involved in stimulus-evoked K^+^ clearance in the rodent hippocampus under approached physiological conditions.
- The Na^+^/K^+^-ATPase is comprised of an α and a β subunit (1:1) and drives uptake of 2 K^+^ from the extracellular space in exchange for 3 Na^+^ from the intracellular compartment. As this transport process translocates both ions against their electrochemical gradients, the Na^+^/K^+^-ATPase relies on ATP hydrolysis as an integral step in its enzymatic cycle. Constant activity of the Na^+^/K^+^-ATPase is required to uphold the transmembrane ionic gradients, which create and maintain the membrane potential. The Na^+^/K^+^-ATPase is, in addition, involved in control of neuronal excitability (Haglund and Schwartzkroin, 1990; Wang et al., 2012; Gulledge et al., 2013) and has been indicated as a key contributor to management of extracellular [K^+^] following activity-induced K^+^ release: Inhibition of the Na^+^/K^+^-ATPase yielded prolonged [K^+^]_o_ recovery times upon electrical stimulation in the rat optic nerve (Ransom et al., 2000) and hippocampus (D'Ambrosio et al., 2002; Larsen et al., 2014, see Figure 2). In support of a dominating role of astrocytes in K^+^ clearance during neuronal activity, astrocytes display a maximal Na^+^/K^+^-ATPase transport activity (V_max_) considerably larger than that of their neuronal counterpart (Kimelberg et al., 1978; Walz and Hertz, 1982; White et al., 1992; Hajek et al., 1996). The astrocytic Na^+^/K^+^-ATPase isoform combination is, in addition, of a composition that allows astrocytes to increase their Na^+^/K^+^-ATPase activity as a function of elevated extracellular [K^+^] (Grisar et al., 1980; Walz and Hertz, 1982; Hajek et al., 1996; Larsen et al., 2014) and thus allows astrocytes to respond specifically to the K^+^ transients observed in connection with neuronal activity. As different isoforms and/or subunit compositions of the Na^+^/K^+^-ATPase, each with distinct kinetic characteristics, are expressed in different cellular structures of the CNS, one may speculate whether these combinations are assigned specific roles in regard to management of activity-induced extracellular K^+^ transients.

This review aims at presenting the CNS localization, role, and physiological impact of the different subunit isoform combinations of the Na^+∕^K^+^-ATPase in the management of extracellular K^+^ in the brain.

## Localization of the Na^+^/K^+^-ATPase isoforms in neurons and astrocytes

**Figure 3:**
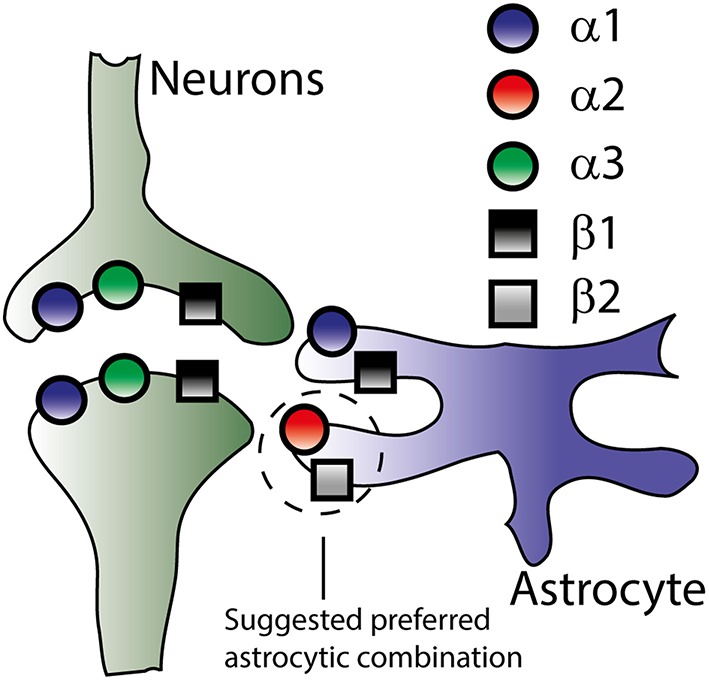
**Na^+^/K^+^-ATPase subunit isoform distribution in neurons and astrocytes**. Though the literature offers no exact distribution of the Na^+^/K^+^-ATPase subunit isoforms (see text), this figure illustrates the overall consensus on isoform-specific expression of the Na^+^/K^+^-ATPase in neurons and astrocytes based on both mRNA and protein expression.

Four isoforms of the α subunit of the Na^+^/K^+^-ATPase have been cloned (α1-α4) and three isoforms of the β subunit (β1-β3) (Blanco, 2005). Of these cloned isoforms, only α1-α3 and β1-β3 have been detected in the mammalian brain (McGrail et al., 1991; Cameron et al., 1994; Martín-Vasallo et al., 2000) although their quantity and cellular distribution remains unsettled and may depend on the brain region. The general picture that emerges reveals mRNA transcripts of α1 and β1 in both neurons and astrocytes while α2 and β2 are detected in astrocytes, and α3 exclusively in neuronal structures (Watts et al., 1991; Li et al., 2013; Zhang et al., 2014, see Figure 3). β3 transcript is prevalent in oligodendrocytes but negligible in astrocytes and neurons (Zhang et al., 2014). The total Na^+^/K^+^-ATPase α subunit mRNA increases about 10-fold from the fetal to adult stage with the α3 transcript reaching maximal and adult levels around post-natal day 7 which is only achieved for α1 and α2 around post-natal day 25 (Orlowski and Lingrel, 1988). The isoform expression pattern obtained at the mRNA level is more or less reflected at the protein level: α1 and β1 are detected in neurons and astrocytes, α2 and β2 predominantly in astrocytes and α3 in neurons (McGrail and Sweadner, 1986; McGrail et al., 1991; Cameron et al., 1994; Cholet et al., 2002; Richards et al., 2007). Alternative expression patterns have been reported which may be due to distinct regional differences in various brain structures and/or developmental stages, subpopulations of neurons with altered isoform preference, altered expression in cultured neurons/astrocytes, and/or non-specific antibodies. Examples include detection of neuronal expression of the α2 isoform in late gestation mouse embryos (Moseley et al., 2003) and in cultures and sections of rat hippocampus (McGrail et al., 1991; Cameron et al., 1994) as well as neuronal expression of β2 in cerebellar sections and co-cultures (Peng et al., 1997).

As opposed to the uniform plasma membrane distribution of α1 in primary cultures of rat hippocampal astrocytes and neurons, α2 and α3 appeared to be organized in a reticular pattern paralleling the underlying endoplasmic reticulum (Juhaszova and Blaustein, 1997). In addition, α2 displayed co-localization with the Na^+^/Ca^2+^ exchanger in the plasma membrane of rat astrocytes (Juhaszova and Blaustein, 1997) and localized to the glial leaflets surrounding dendritic spines (Cholet et al., 2002). The distinct cellular (α1, α3, β1 in neurons and α1, α2, β1, β2 in astrocytes) and subcellular localization pattern underscores the notion of the different Na^+^/K^+^-ATPase isoforms serving distinct physiological roles in the CNS.

## Na^+^ and K^+^ affinities of the neuronal and astrocytic Na^+^/K^+^-ATPase isoform combinations

**Figure 4:**
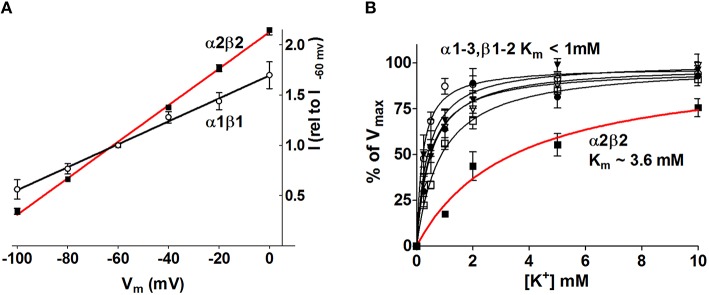
**The Na^+^/K^+^-ATPase activity increases in an isoform-specific manner as a function of [K^+^]_o_ and V_m_**. The Na^+^/K^+^-ATPase-mediated current was determined with two-electrode voltage clamp in *Xenopus* oocytes heterologously expressing the various astrocytic and neuronal subunit isoform combinations. **(A)** The α2β2 Na^+^/K^+^-ATPase displayed the most prominent voltage-sensitivity with increased Na^+^/K^+^-ATPase activity as a function of membrane depolarization. For clarity only α2β2 and α1β1 are illustrated, the other combinations all had intermediate I/V relationships. **(B)** the Na^+^/K^+^-ATPase-mediated current was determined as a function of [K^+^] at V_m_ = −80 mV. The α2β2 combination displayed a low apparent affinity for K^+^, K'_m_ ~3.6 mM, whereas all other tested isoform combinations saturated at lower K^+^ concentrations, K'_m_ < 1.0 mM. Figure modified from Larsen et al. (2014) with permission.

The obvious manner in which the Na^+^/K^+^-ATPase may be exerting isoform-specific roles in neurophysiology may be by the distinct apparent ion affinities (K'_m_) of the different α and β isoform combinations. Of the isoforms expressed in the CNS, all subunit isoform combinations are able to assemble and be catalytically competent in heterologous expression systems (Crambert et al., 2000; Blanco, 2005; Larsen et al., 2014). The apparent ion affinities of the individual isoform combinations have been investigated in a number of cellular and membranous systems with each their advantages and disadvantages. Most commonly employed are leaky membrane preparations (non-sided preparations) obtained from membrane extracts of diverse tissue, mammalian cell lines (i.e., HeLa, COS-1), Sf-9 insect cells, or yeast cells all expressing select Na^+^/K^+^-ATPase isoforms (Sweadner, 1985; Jewell and Lingrel, 1991; Blanco et al., 1995; Therien et al., 1996; Müller-Ehmsen et al., 2001; Toustrup-Jensen et al., 2001), although intact cellular systems (sided preparations), i.e., Na^+^/K^+^-ATPase-expressing HeLa cells or *Xenopus* oocytes have also been employed (Munzer et al., 1994; Zahler et al., 1997; Hasler et al., 1998; Crambert et al., 2000; Horisberger and Kharoubi-Hess, 2002; Larsen et al., 2014). The advantage of the leaky membrane approach is the complete control of all ion concentrations, the experimental ability to address select steps of the Na^+^/K^+^-ATPase transport cycle, and the high degree of accuracy. An inherent disadvantage is the non-physiological ion concentrations facing the two sides of the Na^+^/K^+^-ATPase, which, due to the reciprocal influence of Na^+^ and K^+^ on the other ion's ability to bind, affect the apparent ion affinities of the Na^+^/K^+^-ATPase (Toustrup-Jensen et al., 2001). The transmembrane potential (or lack thereof in isolated membranes) also affects Na^+^/K^+^-ATPase activity and ion affinities in an isoform-specific manner (Larsen et al., 2014, also see Figure 4A). While the apparent K^+^ affinity is uncomplicated to approach in intact cellular systems with physiological ion concentrations and membrane potential, determination of the apparent Na^+^ affinity at the intracellular face of the Na^+^/K^+^-ATPase presents an experimental challenge. For both approaches, expression of the different Na^+^/K^+^-ATPase α isoforms in mammalian cells endogenously expressing β1 (Orlowski and Lingrel, 1988; Therien et al., 1996; Toustrup-Jensen et al., 2001) has prevented detailed analysis of the kinetic impact of β2 and β3 on the different α isoforms. Sf9 insect cells and *Xenopus laevis* oocytes express low levels of endogenous Na^+^/K^+^-ATPase and kinetic analysis of Na^+^/K^+^-ATPase expressed in these cell types have thereby paved the road toward determination of the apparent ion affinities of all isoform combinations (Blanco et al., 1995; Crambert et al., 2000; Larsen et al., 2014). With few studies comparing all, or the majority of, isoform combinations in parallel experimental sessions, a certain diversity exists in the obtained apparent ion affinities of the Na^+^/K^+^-ATPase. The general consensus emerging for the neuronal and astrocytic isoform combinations is as follows: When paired with the ubiquitous β1 isoform, the three α isoforms display similar apparent K^+^ affinities in the 1–2 mM range, irrespective of the sidedness of the membrane preparations (Urayama and Nakao, 1979; Sweadner, 1985; Jewell and Lingrel, 1991; Therien et al., 1996; Crambert et al., 2000; Horisberger and Kharoubi-Hess, 2002; Larsen et al., 2014), although a few studies reported a slightly reduced K^+^ affinity of the α2 isoform (Blanco et al., 1995; Müller-Ehmsen et al., 2001). The apparent Na^+^ affinities of α1 and α2, when in constellation with β1, have been reported at quite a range of values (with K'_m_s centered around 10-16 mM), although roughly similar between the isoforms in each study, with no general trend regarding which of the two displayed a tendency toward higher affinity than the other (Jewell and Lingrel, 1991; Blanco et al., 1995; Zahler et al., 1997; Crambert et al., 2000; Müller-Ehmsen et al., 2001; Horisberger and Kharoubi-Hess, 2002). α3, on the other hand, displayed a much reduced Na^+^ affinity with values centered around K'_m_ of 30 mM, when determined in sided systems with physiological intracellular K^+^ concentrations (Lytton, 1985; Zahler et al., 1997; Crambert et al., 2000; Horisberger and Kharoubi-Hess, 2002; Blanco, 2005). In the leaky membrane preparations, the apparent affinity of α3 toward Na^+^ was either similar or only slightly different from that of α1 (Sweadner, 1985; Jewell and Lingrel, 1991; Therien et al., 1996). Pairing with the astrocytic β2 isoform did not significantly alter the apparent K^+^ affinity of α1 or α3 whereas the α2β2 combination displayed a reduced K^+^ affinity with K'_m_s in the 3-5 mM range (Blanco et al., 1995; Crambert et al., 2000; Larsen et al., 2014, also see Figure 4B) while the apparent Na^+^ affinity of α2 increased to around 7-8 mM upon pairing with β2 (Blanco et al., 1995). The increased apparent Na^+^ affinity may be induced by a β2-dependent shift of the conformational equilibrium toward the E1P state (Hilbers et al., 2016). The isoform-specific relative ion affinities, which are of physiological relevance for clearance of K^+^ from the extracellular space of the central nervous system, are summarized as follows:

$$\begin{array}{l} \text{K}{'}_{\text{m}\left(\text{K}+\right)}:\alpha 1\beta 1\approx \alpha 1\beta 2\approx \alpha 2\beta 1\approx \alpha 3\beta 1<\alpha 2\beta 2\\ \text{K}{'}_{\text{m}\left(\text{Na}+\right)}:\alpha 1\beta 2\approx \alpha 2\beta 2<\alpha 1\beta 1\approx \alpha 2\beta 1<<\alpha 3\beta 1 \end{array}$$

## The physiological role of the Na^+^/K^+^-ATPase isoform combinations in K^+^ clearance

The diverse roles of the Na^+^/K^+^-ATPase in management of [K^+^]_o_ in the central nervous system suggest a scenario in which explicit subunit isoform combinations perform distinct temporal and spatial roles. During the neuronal stimulus, the astrocytic Na^+^/K^+^-ATPase α2β2 combination appears specifically geared to respond to the resultant increase in extracellular [K^+^] due to its low apparent K^+^ affinity: With saturation of all other isoform combinations at basal [K^+^]_o_, an affinity constant of α2β2 in the 3-4 mM range allows this isoform combination to increase its transport activity when faced with a K^+^ load and thus increase the rate of K^+^ clearance from the extracellular space and into the nearby astrocytes (Larsen et al., 2014, Figure 4B). In addition, the membrane depolarization brought about by the increased extracellular [K^+^] promoted enhanced transport activity of all isoform combinations although the voltage-sensitivity was most pronounced for the α2β2 combination (Larsen et al., 2014, Figure 4A). The select glial expression of the Na^+^/K^+^-ATPase α2β2 isoform combination (McGrail et al., 1991; Cameron et al., 1994; Zhang et al., 2014) and localization of α2 to the glial processes surrounding neuronal dendrites (Cholet et al., 2002) taken together with the voltage- and K^+^-sensitivity of the α2β2 isoform combination, renders it an efficient responder to stimulus-evoked K^+^ transients in the extracellular space and thus an important contributor to management of the extracellular K^+^ during neuronal activity.

Given the nature of the Na^+^/K^+^-ATPase, the transport activity can, in addition to its K^+^-dependent activation, also be governed by activity-dependent fluctuations in the intracellular Na^+^ concentration. As neurons propagate action potentials with subsequent synaptic activity, neuronal Na^+^ accumulation occurs gradually via ligand- and voltage-gated Na^+^ channels in parallel to the release of K^+^ to the extracellular space (Langer and Rose, 2009). This [Na^+^]_i_ increase prompts enhanced activation of the neuronal Na^+^/K^+^-ATPase of the isoform combination α3β1, with its characteristically low apparent Na^+^ affinity (Lytton, 1985; Zahler et al., 1997; Crambert et al., 2000; Horisberger and Kharoubi-Hess, 2002; Blanco, 2005). The α3β1-mediated extrusion of Na^+^ and uptake of K^+^ permits the neurons to reestablish the concentration gradients of the two ions following the neuronal activity and the subsequent gradual release of the astrocytic K^+^ stores via Kir4.1 (Chever et al., 2010). In this way the neuron is ready to propagate a succeeding volley of action potentials. Since the α3β1-mediated activity is governed primarily by the intracellular Na^+^ concentration, its enhanced activity continues until normalization of [Na^+^]_i_ has occurred. As this process occurs toward the termination of the neuronal activity (Grafe and Ballanyi, 1987) and is independent of the [K^+^]_o_, it may lead to an undershoot of [K^+^]_o_ in which the concentration drops below baseline in the later phase of the stimulus-evoked K^+^ transient prior to its stabilization (Ransom et al., 2000; D'Ambrosio et al., 2002).

**Figure 5:**
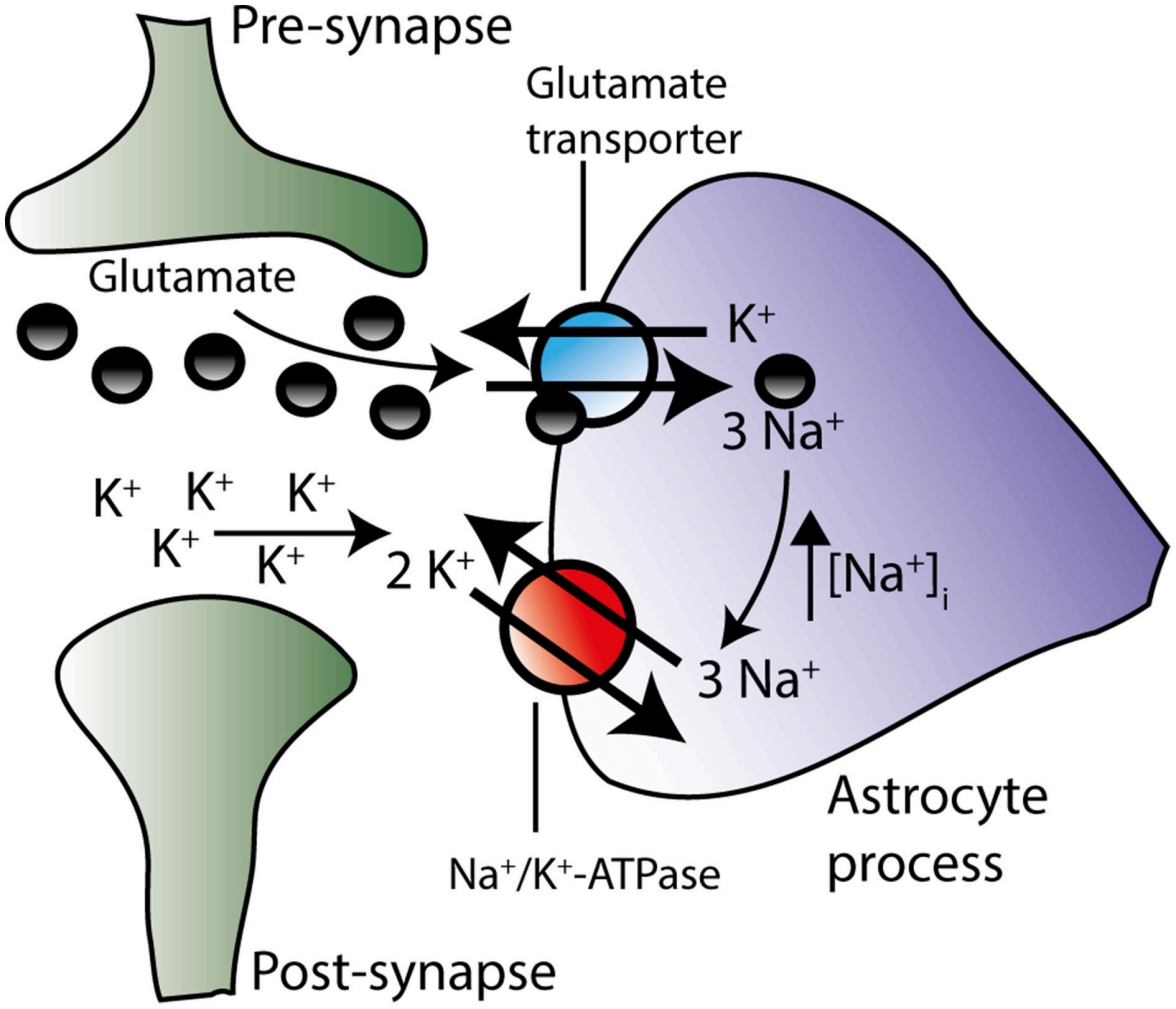
**The Na^+^/K^+^-ATPase is suggested to work in concert with the glutamate transporter**. During action potential propagation, glutamate and K^+^ are both released from the pre-synaptic neuron. Glutamate-induced depolarization of the post-synaptic neuron instigates further K^+^ release. By diffusion glutamate and K^+^ reach the peri-synaptic astrocyte processes located in close proximity to the synapse. Glutamate is transported into the astrocyte along with Na^+^ and in exchange for K^+^ (see text for stoichiometry, H^+^ transport has been omitted on figure for clarity). The resultant rise in intracellular Na^+^ may trigger the activation of the Na^+^/K^+^-ATPase isoform combinations, which are not already saturated at their Na^+^ binding site. Increased activity of the Na^+^/K^+^-ATPase would result in enhanced extrusion of the accumulated Na^+^ and, concomitantly, in amplified removal of extracellular K^+^ in the synaptic area. Although this proposal (based on co-localization patterns and prolonged glutamate exposure of astrocytic cultures) remains to be verified in brain slices during neuronal activity, these two transporters may work in concert in order to efficiently clear both glutamate and K^+^ from the extracellular space.

Glutamatergic signaling represents the majority of the excitatory stimulus in the central nervous system (Danbolt, 2001). Upon vesicular release of glutamate into the synaptic cleft, glutamate is swiftly removed from the extracellular space by Na^+^-coupled glutamate transporters primarily located in adjacent astrocytes (Bergles and Jahr, 1997; Danbolt, 2001). Of the five cloned isoforms of the glutamate transporter (Excitatory Amino Acid Transporters, EAAT1-5), astrocytes express EAAT1 and EAAT2, with the latter being the dominantly expressed isoform in hippocampal tissue (Bar-Peled et al., 1997; Furuta et al., 1997; Lehre and Danbolt, 1998) and estimated to account for around 90% of the glutamate uptake in the mammalian brain (Danbolt et al., 1992). During one transport cycle, inward translocation of one glutamate is driven by cotransport of three Na^+^ and one H^+^ while one K^+^ is extruded (Levy et al., 1998). Astrocytic Na^+^ accumulation thus occurs upon glutamate transporter activity in cultured astrocytes (Chatton et al., 2000; Illarionava et al., 2014) and acute brain slices (Langer and Rose, 2009; Langer et al., 2012). The astrocytic glutamate transporters display some degree of co-localization with the Na^+^/K^+^-ATPase α2 in glial leaflets surrounding glutamatergic synapses (Cholet et al., 2002; Rose et al., 2009; Rose and Karus, 2013), suggesting functional interplay between these two transport proteins, as illustrated in Figure 5. Indeed, in cultured astrocytes, inhibition of the Na^+^/K^+^-ATPase instantly compromised glutamate uptake (Rose et al., 2009; Bauer et al., 2012; Illarionava et al., 2014) while Na^+^ accumulation via glutamate transporter activity (or mimicking thereof via a Na^+^ ionophor) stimulated Na^+^/K^+^-ATPase-mediated K^+^ uptake driven by the α2 isoform (Pellerin and Magistretti, 1997; Bender et al., 1998; Munhoz et al., 2005; Sheean et al., 2013). However, this glutamate transporter-mediated activation of Na^+^/K^+^-ATPase activity in cultured cells was observed exclusively upon prolonged (up to 20 min) exposure to glutamate (Pellerin and Magistretti, 1997; Bender et al., 1998; Munhoz et al., 2005; Sheean et al., 2013) which could suggest an indirect activation of the Na^+^/K^+^-ATPase (Munhoz et al., 2005) rather than a direct effect of a Na^+^ transient. It therefore remains to be resolved if the glial glutamate transporter-mediated Na^+^ transients occurring during brief neuronal activity (Langer and Rose, 2009) directly activates the glial Na^+^/K^+^-ATPase from the cytosolic face of the membrane. For the Na^+^/K^+^-ATPase to respond to stimulus-evoked intracellular Na^+^ transients, it follows that the intracellular face of the transport protein is not already saturated at the basal glial Na^+^ concentration of ~12 mM (Langer et al., 2012). With the above-mentioned challenges of recording the intracellular Na^+^ affinity of various isoform combinations of the Na^+^/K^+^-ATPase in sided preparations of intact cells, especially in regard to β2, it remains to be revealed which glial isoform combination(s) may display Na^+^ affinities in a range which would allow for increased activity when faced with stimulus-induced intracellular Na^+^ transients.

It follows that the kinetic behavior and therefore physiological specialization of the different α isoforms may depend on the interacting β subunit and the physiological contribution of the different isoforms hinges on their quantitative expression in the different cellular structures of the CNS. Little is known about the quantitative distribution of the different isoforms at the protein level (due to challenges with comparison between different antibodies with different antigen specificity) as is their favored accessory subunit combinations, although α2 has been shown to favor interaction with the β2 subunit in mouse brain extracts (Tokhtaeva et al., 2012).

## Involvement of Na^+^/K^+^-ATPase α2 and α3 in central nervous system disease

Alterations in expression of the Na^+^/K^+^-ATPase isoforms and/or dysfunctional mutant forms of the Na^+^/K^+^-ATPase may cause disturbances in the K^+^ homeostasis and therefore affect a range of cellular parameters, i.e., membrane potential, synaptic signaling, rate of activity-evoked K^+^ clearance, glutamate re-absorption etc. Underscoring the importance of these transport proteins for normal physiology, mice with homozygous deletion of the genes *ATP1A2* or *ATP1A3* (encoding α2 and α3) die immediately after birth (Ikeda et al., 2004, 2013). Severe neurological diseases thus arise following various mutations in *ATP1A2* and *ATP1A3* (de Vries et al., 2009; Benarroch, 2011; Bøttger et al., 2012): Familial hemiplegic migraine type 2 (FHM2) arises upon mutations in *ATP1A2* (De Fusco et al., 2003) and is a severe autosomal form of migraine with aura associated with hemiparesis and sometimes accompanied by manifestations such as epilepsy, seizures, ataxia and developmental disabilities (Pietrobon, 2007; Bøttger et al., 2012). A variety of different mutations in α2 has been detected in patients suffering from FHM2 and knock-in mice expressing FHM2-derived mutant forms of α2 have been established and found to mimic FHM2-relevant disease traits (Leo et al., 2011; Bøttger et al., 2016 and reviewed elsewhere in this special issue by Lykke-Hartmann and colleagues): The heterozygous α2(+/W887R) knock-in mouse model displayed a lower induction threshold for cortical spreading depression (a phenomenon observed in association with migraine with aura) and an increased rate of propagation (Leo et al., 2011) whereas the α2(+/G301R) mouse model displayed mood depression, obsessive compulsive disorder, reduced regeneration after cortical spreading depression in males, as well as reduced glutamate uptake in astrocytic cultures obtained from embryonic homozygotes (Bøttger et al., 2016). A selection of the mutations found in human FHM2 patients have been generated *in vitro* to determine the functional impact on the Na^+^/K^+^-ATPase activity. Diverse kinetic implications arise as a consequence of the mutations, e.g., loss-of-function, reduced catalytic turnover, shifted E1↔E2 steady-state conformational equilibrium, increased/decreased apparent K^+^ affinity and increased/decreased apparent Na^+^ affinity (the latter associated with altered basal [Na^+^]_i_) (De Fusco et al., 2003; Segall et al., 2004, 2005; Schack et al., 2012; Toustrup-Jensen et al., 2014). The diverse functional outcome of these mutations, all giving rise to the same disease, underscores the complexity of the role of α2 in Na^+^ and K^+^ management in the central nervous system. However, it remains puzzling why such diverse alterations, e.g., oppositely directed shifts in K^+^ affinity, disrupt neuronal signaling in a manner that gives rise to similar phenotypic outcome. Alterations in Na^+^/K^+^-ATPase α2 isoform expression have been indicated in several neuropathological situations: (i) migraine (with aura) patients displayed altered Na^+^/K^+^-ATPase isoform expression toward versions with higher ouabain sensitivity (Scarrone et al., 2007), (ii) reduced K^+^ affinity of partially purified Na^+^/K^+^-ATPase from neocortices of human epileptic patients (Guillaume et al., 1991), (iii) reduced K^+^ clearance rates observed in cortex of epileptic monkeys (Lewis et al., 1977), and (iv) glial fractions obtained from cats following freezing lesion-induced epilepsy lost their characteristic K^+^-activation, suggesting that α2 and/or β2 are down regulated during the time between the freezing lesion and the occurrence of the epileptogenic states (Grisar et al., 1983). Oppositely, ciliary neurotrophic factor up regulated glial α2 mRNA with subsequent enhanced K^+^ uptake and increased threshold for spreading depolarization (Seidel et al., 2015).

Mutations in the gene *ATP1A3* encoding the α3 isoform have been discovered in patients with rapid-onset dystonia parkinsonism (RDP), alternating hemiplegia of childhood (AHC), and early life epilepsy (de Carvalho Aguiar et al., 2004; Bøttger et al., 2012; Heinzen et al., 2014; Sweney et al., 2015). Expressional analysis of α3 demonstrated the presence of this subunit in anatomical regions correlating with RDP symptoms (Bøttger et al., 2011). Mice with heterozygous deletion of *ATP1A3* displayed movement abnormalities partly in line with the human RDP phenotype (Ikeda et al., 2013; Sugimoto et al., 2014) and were more susceptible to stress-induced depression-like phenotypes (Kirshenbaum et al., 2011). Mice expressing mutant forms of α3 exhibit a phenotype comparable to that of patients with AHC and display hyperexcitability, K^+^-induced spreading depolarization of longer duration, and enhanced seizure susceptibility (Clapcote et al., 2009; Hunanyan et al., 2015; Kirshenbaum et al., 2015, reviewed in detail elsewhere in this special issue by Lykke-Hartmann and colleagues). Select human mutations in *ATP1A3* have been established *in vitro* and the kinetic alterations of the mutated α3 isoforms ranged from loss of function to reduced activity and modified cation binding (Einholm et al., 2010; Toustrup-Jensen et al., 2014; Weigand et al., 2014).

## Conclusion

The Na^+^/K^+^-ATPase exhibits an isoform-specific expression pattern in the mammalian CNS. The distinct kinetic properties of the different catalytic α isoforms hinge, in part, on the accessory β subunit with which they pair and determine their temporal and spatial quantitative contribution to management of the extracellular K^+^ transients occurring in the wake of neuronal activity. While the astrocytic α2 isoform appears specifically geared to respond to the activity-evoked K^+^ transients upon pairing with β2, the excessively low apparent Na^+^ affinity of the α3 ensures neuronal re-uptake of K^+^ once the neuronal activity is terminated. Mutations in the genes encoding the neuronal α3 or the astrocytic α2 give rise to a range of severe neuronal pathologies, although the exact mechanism coupling each point mutation to a distinct disease pattern remains unresolved.

## Author contributions

All authors listed have made substantial, direct and intellectual contribution to the work, and approved it for publication.

## Funding

The laboratory is funded by grants from the Lundbeck Foundation, Danish Medical Research Council and Thorberg's Foundation.

### Conflict of interest statement

The authors declare that the research was conducted in the absence of any commercial or financial relationships that could be construed as a potential conflict of interest.
